# Application of the integrated airway humidification device enhances the humidification effect of the rabbit tracheotomy model

**DOI:** 10.1515/biol-2022-0825

**Published:** 2024-02-08

**Authors:** Honglan Sheng, Jie Ni, Feng Zhao, Mi Tian, Yuhang Zhao, Longmei Dai, Ting Li, Yun Xue, Zongze Song, Qiong Yu

**Affiliations:** Department of Anaesthesiology, Huashan Hospital, Fudan University, Shanghai 200040, China; Department of Critical Care Medicine, Huashan Hospital, Fudan University, Shanghai 200040, China; Department of Integrative Medicine, Huashan Hospital, Fudan University, Shanghai 200040, China; Department of Critical Care Medicine, Shanghai Sixth People’s Hospital Affiliated to Shanghai Jiao Tong University School of Medicine, Shanghai 200000, China

**Keywords:** tracheotomy, airway humidification, animal model, inflammatory response, histopathology

## Abstract

Long-term mechanical ventilation after tracheotomy is a common treatment in intensive care unit patients. This study investigated the differences among the effects of different wetting states on the airway, lung, and serum inflammatory factors. New Zealand rabbits (*n* = 36) were selected to construct tracheotomy models and then divided into four groups: Model, Mask, YTH, and Sham groups. Lung tissue dry/wet ratio was used to evaluate the humidification effect; cytokines, including tumor necrosis factor-α, interleukin (IL)-6, IL-8, and IL-10, were used to evaluate the inflammatory response; hematoxylin and eosin staining was used to evaluate the histopathology. Post hoc analysis based on the Dunnett *t*-test was applied. A self-developed integrated wetting device could increase the utilization of wetting solution, enhance the effect of wetting to protect tissue integrity, and suppress airway inflammation, reducing the expression of pro-inflammatory factors while promoting the expression of anti-inflammatory factor IL-10 to inhibit the inflammatory response, compared to other methods. The integrated humidification device provided a new method for clinical nursing practice, improving clinical efficiency and reducing nursing workload. Further clinical trials are required to test its effectiveness and safety in the clinic.

## Graphical abstract

**Figure:**
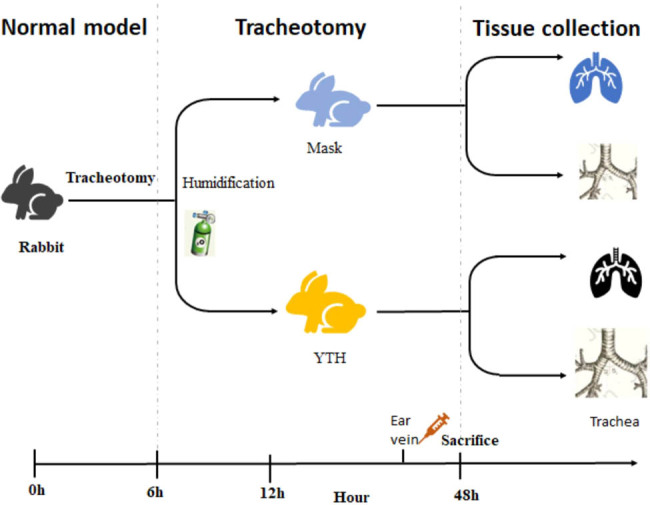

## 1 Introduction

Tracheotomy is a procedure during which a tracheal tube is placed in the trachea through an incision in the anterior tracheal wall to construct an artificial airway. Long-term mechanical ventilation based on tracheotomy has been an important treatment for critically ill patients. Also, early tracheotomy has been associated with reduced mortality and pneumonia rates in patients with long-term follow-up [1,2]. However, direct airway opening in medium- and long-term tracheotomy patients leads to airway water loss, resulting in airway dryness and sputum stickiness, and may even lead to sputum blockage, causing hypoxia and asphyxia. The incidence of tracheal tube occlusion after tracheotomy was reported to be 14–43% [3]. Moreover, tracheotomy patients can lose more than 800 ml of airway water daily. In addition, the inflammatory condition may appear in the airway post-tracheotomy [4]. Besides, prolonged tracheotomy increases the possibility of complications, including pneumothorax, bleeding, infection, intubation obstruction, and fistula formation [5,6]. The intact upper respiratory tract protects the inhaled air by adsorbing and removing foreign bodies and pathogens. Meanwhile, the airway epithelium warms and humidifies the inhaled air under physiological conditions. On the contrary, tracheotomy deprives patients of the warming and humidifying effect of the airway on inhaled oxygen. Therefore, direct inhalation of cold and dry oxygen in patients with assisted breathing may damage the airway, resulting in decreased body temperature, dyspnea, and airway obstruction [7,8]. In addition, ventilator-associated pneumonia is the leading cause of death in patients equipped with invasive mechanical ventilation, accounting for 25% of infections in intensive care unit wards [9,10].

Airway humidification can reduce mortality and pulmonary infections in patients with assisted ventilation [11]. There are several types of humidification devices in clinical use, including oxygen atomizers (OA), heat and moisture exchangers (HME), and heated humidifiers (HH) [12]. Fogging devices can be divided into Jet and ultrasonic nebulizers according to their principle. Jet nebulizers are based on the venturi principle, while ultrasonic nebulizers utilize the inverse piezoelectric effect to convert alternating current into high-frequency acoustic energy to atomize liquid water [13,14]. A clinical study compared the effects of OA, HH, and HME in patients with severe traumatic brain injury and suggested that HH and HME are superior to OA in reducing the incidence of infection; however, OA relieved the care-related burden [12]. In addition, other studies reported the difference in effectiveness between HH and HME wetting. A meta-analysis of HH on wetting in adults and children reported no significant difference between the effect of HH and HME on the incidence of airway obstruction, pneumonia, and death. However, some evidence indicated that hydrophobic HME prevents the incidence of pneumonia better than the other [10,15].

During mechanical ventilation, if bypassing the upper airway, manual humidification is necessary in case the cold and dry oxygen causes secondary damage by directly injuring the airway epithelium [16,17]. Airway humidification is also mandatory, especially for patients with nasopharyngeal and pulmonary diseases or postoperative and neurological disorders. Depending on the degree of airway humidification, patients can present with symptoms from inadequate humidification (such as hardly absorbable mucous sputum and crackling breath sounds) to appropriate humidification (such as moderate volume of thin sputum that is easily absorbed and clear airway) to excessive humidification (such as large volume of foamy sputum and dyspnea) [12]. However, existing studies rarely addressed the effect of the degree and duration of humidification on airway and lung inflammation.

This study investigated the degree of wetting by different devices for inhaled oxygen wetting in a well-studied rabbit tracheotomy model [18] to establish the differences among the effects of different wetting states on the airway and lung and serum inflammatory factors.

## 2 Materials and methods

### 2.1 Animal models

Thirty-six New Zealand rabbits weighing 2.5–3.0 kg were purchased from the Shanghai Veterinary Research Institute, Chinese Academy of Agricultural Sciences [license number: SCXK (Shanghai) 2022-0027] and housed in the animal house of Shanghai Academy of Agricultural Sciences at 25 ± 2°C, 50 ± 15% humidity, 12 h light–dark cycle, with free access to food and water. All animal studies were done in compliance with the regulations and guidelines of Shanghai Medical College of Fudan University institutional animal care and conducted according to the AAALAC and the IACUC guidelines (ethics number: 20200615S).

These 36 rabbits were randomly divided into four groups (*n* = 9/group): sham operation group (Sham), model group (Model), mask group (Mask), and integrated device group (YTH). Each group was then randomly divided into three subgroups depending on the time (6, 12, and 48 h; *n* = 9/group). Rabbits in the Model, Mask, and YTH subgroups received tracheotomy and airway humidification, while rabbits in the Sham group underwent sham surgical operation without airway wetting. After reaching the wetting time, each subgroup’s blood was collected via a vein at the ear margin. Then, euthanasia and rapid tracheal and lung tissue extraction were performed. The blood was undisturbed, kept at room temperature for 2 h, and then centrifuged at 3500*g* at 4°C for 20 min. The serum was frozen at −80°C. After removal, the trachea was quickly fixed in 4% paraformaldehyde to prepare hematoxylin and eosin (HE)-stained pathological sections. The wet and dry weight of the lung tissues were measured respectively, and ultimately, the wet-to-dry ratio was calculated.


**Ethical approval:** The research related to animal use has been complied with all the relevant national regulations and institutional policies for the care and use of animals, and has been approved by the Ethics Committee of Shanghai Medical College of Fudan University (ethics number: 20200615S).

### 2.2 Tracheotomy and humidification

The rabbit was fasted for 12 h before surgery and then anesthetized using dexmedetomidine at a dose of 60 µg/kg (Yangtze River Pharmaceutical Group Co., Ltd., lot no. 22011731). After the pain reaction and corneal reflexes disappeared, the rabbit was fixed in a supine position. The skin was prepared, a cavity towel was laid, and the area was disinfected three times. A longitudinal incision of approximately 1 cm was then made along the anterior midline and 2 cm above the superior sternal recess, and the trachea was exposed after bluntly separating the muscular layer by layer. An inverted T-shaped incision was then performed under the thyroid cartilage, where a homemade rabbit tracheal cannula was inserted. The cannula was fixed with surgical thread, and the muscle layers and skin were successively sewn. Next, erythromycin ointment was applied to the wound to prevent the infection. In the sham-operated group, the skin was incised, the trachea was exposed after anesthesia, and the muscle layers and skin were sutured afterward. Postoperatively, wound bleeding and breathing were closely observed to avoid asphyxia due to body position.

Oxygen-driven continuous airway wetting (0.45% NaCl solution, oxygen flow rate 4 L/min) was selectively performed in different groups after the operation. Model and Sham groups received no airway wetting, while oxygen mask wetting was used in the Mask group and oxygen-supplied wetting with an integrated device in the YTH group. Sputum was aspirated from all rabbits in the Model, Mask, and YTH groups after each humidification session. Sputum consistency criteria were as follows [19]: Degree I (thin sputum): sputum was abundant and thin, coughs were frequent, wheeze rale could be heard in the airway, and no sputum was detected on the inner wall of the aspiration tube after aspiration; Degree II (moderate mucous sputum): the appearance of sputum was thicker than Degree I, no dry rales were heard when pulmonary auscultation, and a small amount of sputum was detected on the inner wall of the aspiration tube after aspiration which could be easily flushed out by water; Degree III (severe mucous sputum): the appearance of sputum was sticky, yellow, and accompanied by blood crust, and it was not easy to be aspirated or coughed out.

### 2.3 Pulmonary dryness and humidity

The pulmonary wet-to-dry ratio was one of the indicators used to evaluate the effect of wetting quantitatively. Fresh lung tissue was removed quickly from rabbits after euthanasia. After using filter paper to aspirate surface water, direct weight was defined as the wet lung weight. The lung tissue was subsequently baked in an oven at 80°C for 1 week to dry the tissue moisture, and then the dry lung weight was weighed. The lung dry-to-wet ratio was calculated using the following formula: lung dry weight/lung wet weight × 100%.

### 2.4 Enzyme-linked immunosorbent assay (ELISA)

Tissue specimens were taken from both ends and the central part of the main trachea (from 2 cm below the tracheal intubation and two portions of the hilar area). Five specimens were placed in a 10% formaldehyde solution for pathological examination. After 48 h, 2 ml of venous blood was drawn from the marginal ear vein and centrifuged at 3000 rpm for 20 min. The supernatant was divided into EP tubes and stored at −80°C in a refrigerator. Kits of tumor necrosis factor-α (TNF-α) (Cloud-Clone SEA133Rb, L210507091), interleukin (IL)-6 (Cloud-Clone SEA079Rb, L210506259), IL-8 (Cloud-Clone SEA080Rb, L210507080), and IL-10 (Cloud-Clone SEA056Rb, L210507084) were used to detect inflammatory factors in rabbit serum and evaluate the inflammation index. The standard curves were performed according to the instructions of the kits. Ultimately, the absolute levels of TNF-α, IL-6, IL-8, and IL-10 in each group were calculated according to the absorbance of the samples.

### 2.5 Histopathology

Tracheal tissues (3 cm in diameter) were selected from New Zealand rabbits, 1 cm below the tracheal ligation line. The tissues were then fixed with 4% paraformaldehyde for 48 h, paraffin-embedded to prepare wax blocks, sectioned, dewaxed, stained, dehydrated, and sealed to construct stained tissue slices. The slices were observed and photographed under a microscope (Nikon). The tracheal histopathology scoring criteria [20] are shown in Table 1. Tracheal histopathology score = 3 A + B + C + D.

**Table 1:** Tracheal histopathology scoring criteria

Score	Pseudostratified ciliated columnar epithelium (PCCE)	Microglandular hyperplasia (MH)	Intracavitary secretion (IS)	Inflammatory cell infiltration (ICI)
0	Orderly, neat, without missing	None	Little	Not infiltrated
1	Slightly disordered, cilia vary in length	A little	Increased	A little infiltration in the lumen or submucosa
2	Significantly disordered, intermittent	Much	Much with a formed element	Multiple infiltrations of inflammatory cells
3	Necrosis and detachment, submucosal tissue exposed	Much with secreta	Significantly increased	

### 2.6 Statistical analysis

Statistical analysis was performed using GraphPad Prism 7.0. The rank sum test was used to compare dryness and pathology scores among groups. Inflammatory factors were compared by analysis of variance (ANOVA). Multiple comparisons with the model group as a control were performed using post hoc analysis based on the Dunnett *t*-test. *P*-values <0.05 were considered statistically significant.

## 3 Results

### 3.1 Animal model construction

The mean preoperative weight of the experimental rabbits was 2.48 ± 0.14 kg. A total of 27 New Zealand rabbits received tracheotomy and intubation, while 9 received sham surgery. No intraoperative anesthetic death or hemorrhage occurred, and there was no significant dyspnea or poor sputum evacuation after anesthesia and aspiration.

### 3.2 Effect of airway wetting with different devices on sputum viscosity and pulmonary dryness and humidity

Consistent with clinical practice, we performed aspiration on the Model and Mask groups after airway wetting treatment to ensure that post-wetting sputum and softened sputum crusts would not block the airway and interfere with normal breathing. Overall, the sputum in the YTH and Mask groups was clear and thin, easily aspirated, occasionally with yellow sputum and crusts, and it hardly affected breathing. In contrast, in the Model group, the sputum was sticky with sputum and blood crusts; the sputum was not easy to aspirate. Also, respiratory distress with phlegm sound was detected in some rabbits in the Model group. Moreover, the tracheal dissection after the euthanasia of rabbits in each group revealed that the trachea of groups treated with wetting was cleaner, with fewer secretions blocking the airway. Also, the effect of the YTH device-wetting group was superior to that of the Mask wetting group. As for the Model group, the viscosity of sputum and crust formation in the airway gradually increased with time. In 48-h subgroups, beanbag-like sputum accumulation with blood oozing was observed in the main trachea, seriously affecting airway patency. These data indicate that airway humidification is an important nursing regimen for sustained airway patency and sputum removal after tracheotomy and that adequate humidification is more likely to maintain airway ventilation, which improves prognosis.

**Figure 1:**
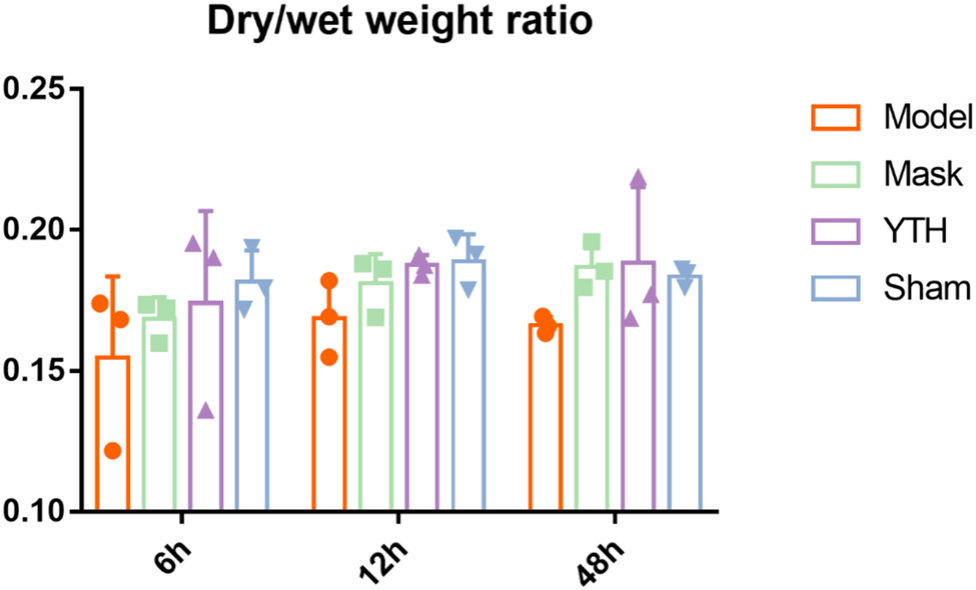
Lung wet-to-dry ratio in rabbits exposed to different wetting methods. The wet-to-dry ratio of whole lungs in different groups at different time points. The relative water content of lung tissue was used to represent the wetting effect. The results of each group are expressed as *x* ± *s* (*n* = 3). A rank sum test was used to compare the difference between the Model group and other groups. *P < 0.05.

By weighing the whole lung tissues of rabbits with dry and wet weights, the wet-to-dry ratio of the lungs was calculated to evaluate the degree of pulmonary tissue dehydration and the wetting effect of different devices. The lung wet-to-dry ratio of rabbits in the Sham group remained high from 6 to 48 h, while it decreased in the Model group, suggesting a lack of water in the pulmonary tissues (Figure 1). The mask and the integrated device had a wetting effect and increased the lung wet-to-dry ratio. The Mask group gradually increased the lung wet-to-dry ratio from 6 to 48 h and reached the normal level at 48 h, while the YTH group achieved a better wetting effect at 12 h, basically reaching the normal level. As depicted in Figure 1, the YTH group had a higher lung wet-to-dry ratio than the Mask group at 6 and 12 h, indicating that the integrated device has a better wetting effect at an early stage and could reach normal levels more quickly. However, the data analysis was not statistically significant, which may be due to the small sample size at each time point and the strict rank sum test method. At 48 h, the lung wet-to-dry ratio was maintained at an approximately normal level in all three groups except the Model group, indicating the improvement of pulmonary dehydration.

### 3.3 Effect of different wetting devices on serum inflammatory factors

**Figure 2:**
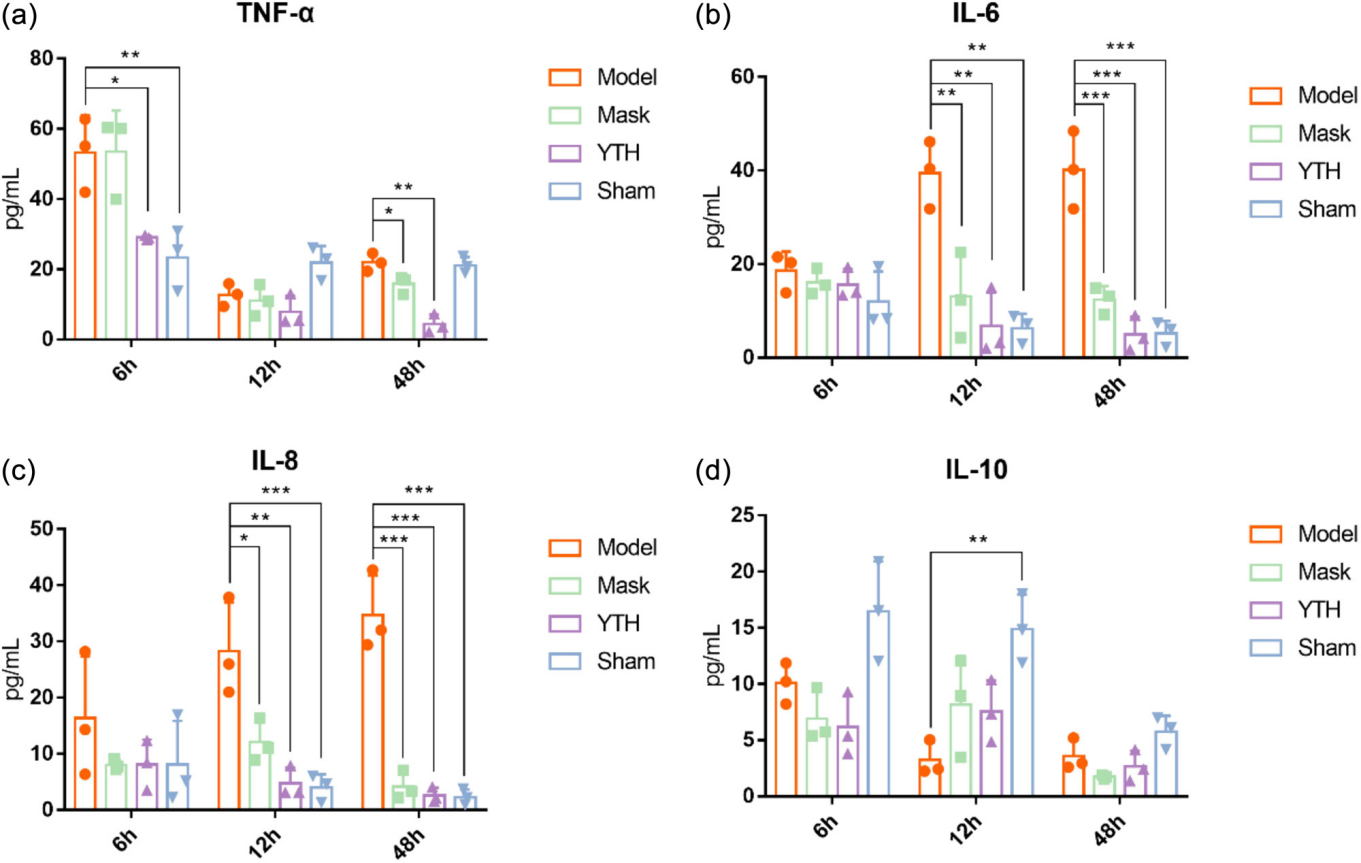
Effect of different wetting methods on serum inflammatory factors. The effect of different wetting methods on serum validation factors was detected by ELISA. a, b, c, and d show changes in serum TNF-α, IL-6, IL-8, and IL-10 levels in each group, respectively. The results were expressed as *x* ± *s* (*n* = 3). ANOVA was used to compare the differences. *P < 0.05, **P < 0.01, and ***P < 0.001.

We obtained the expression of serum inflammatory factors at different times in rabbits by ELISA. In the Model group, serum TNF-α levels significantly increased at 6 h compared with the Sham group (Figure 2a, *P* < 0.01) and decreased to normal levels after 12 h. In the Sham group, serum TNF-α levels were significantly decreased at 48 h (Figure 2a, *P* < 0.01) compared with the Model group. In addition, the TNF-α level was significantly lower in the Mask group compared with the Model group at 48 h (Figure 2a, *P* < 0.05) but remained unchanged from 6 to 12 h. The YTH group showed a significant decrease in serum TNF-α levels at 6 h (Figure 2a, *P* < 0.05) and a decreasing trend at 48 h.

The pro-inflammatory factors IL-6 and IL-8 in the Model group increased from 12 to 48 h (Figure 2a, all *P* < 0.01). In the Mask and YTH groups, IL-6 and IL-8 decreased at 12 h (Figure 2b and c, Mask IL-6 *P* < 0.01, YTH IL-6 *P* < 0.01, Mask IL-8 *P* < 0.05, YTH IL-8 *P* < 0.01) and 48 h (Figure 2b and c, Mask IL-6 *P* < 0.001, YTH IL-6 *P* < 0.001, Mask IL-8 *P* < 0.001, YTH IL-8 *P* < 0.001). In addition, Figure 2a and b suggests that the integrated device had an enhanced inhibitory effect on IL-6 and IL-8 expression than the Mask group. Figure 2d shows that tracheotomy decreased the expression of anti-inflammatory cytokines, and the difference between the Model and Sham groups reached its maximum at 12 h (*P* < 0.05). IL-10 expression was higher in the YTH and Mask groups than in the Model group but with no statistical significance (*P* > 0.05). These results suggest that inadequate post-tracheotomy wetting might increase the expression of pro-inflammatory factors, while an integrated device suppresses airway inflammation.

### 3.4 Histopathological effects of different airway wetting devices

**Figure 3:**
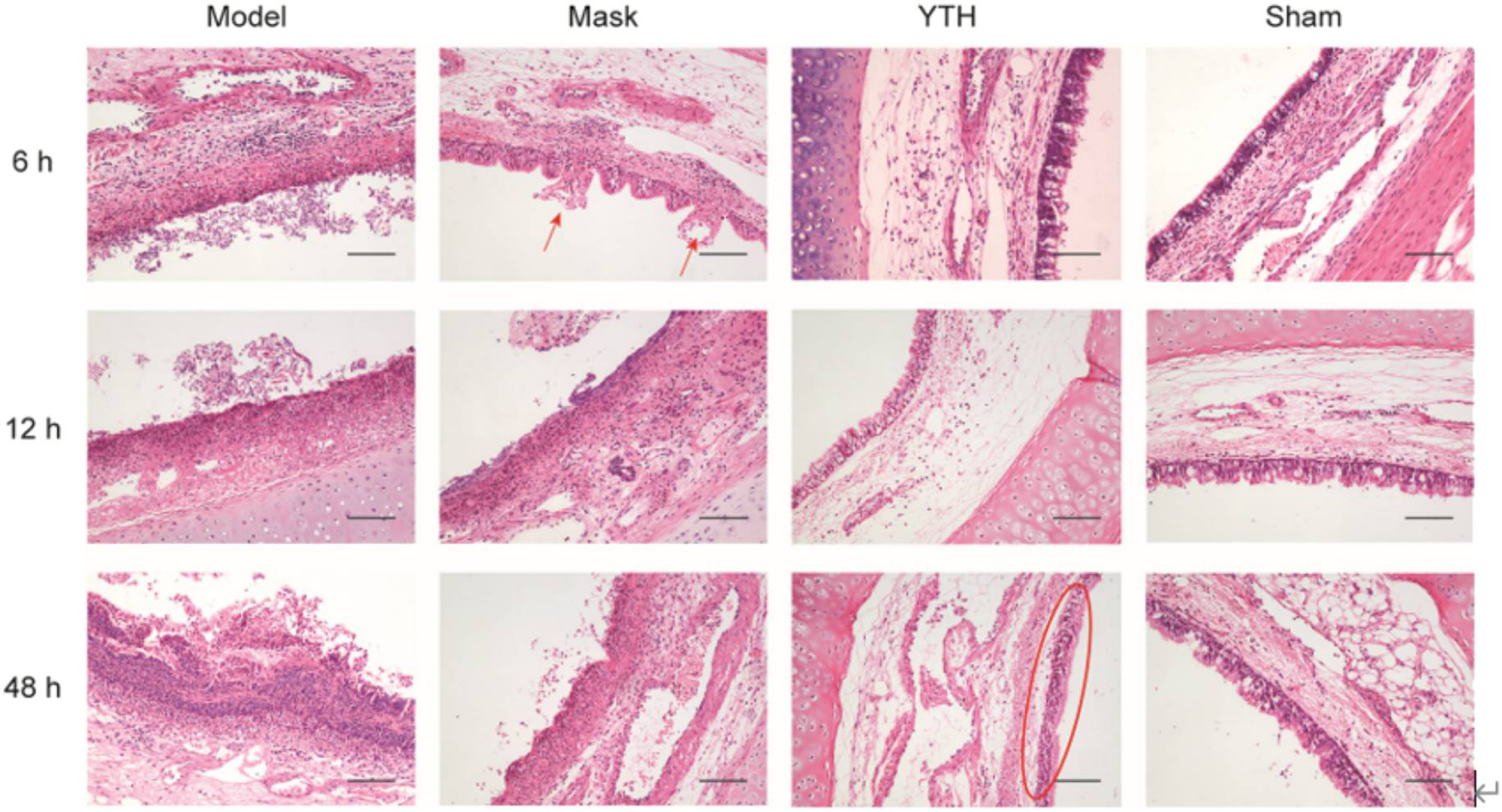
Pathological changes of rabbit trachea under different wetting methods. Morphological changes (HE-stained sections) of rabbit trachea in different groups at different time points. The YTH group maintained the structural integrity of the airway epithelium at 48 h (marked by ellipses). The Mask group showed structural disruption of the airway epithelium at 6 h (marked by arrows). Scale bar: 100 μm.

**Figure 4:**
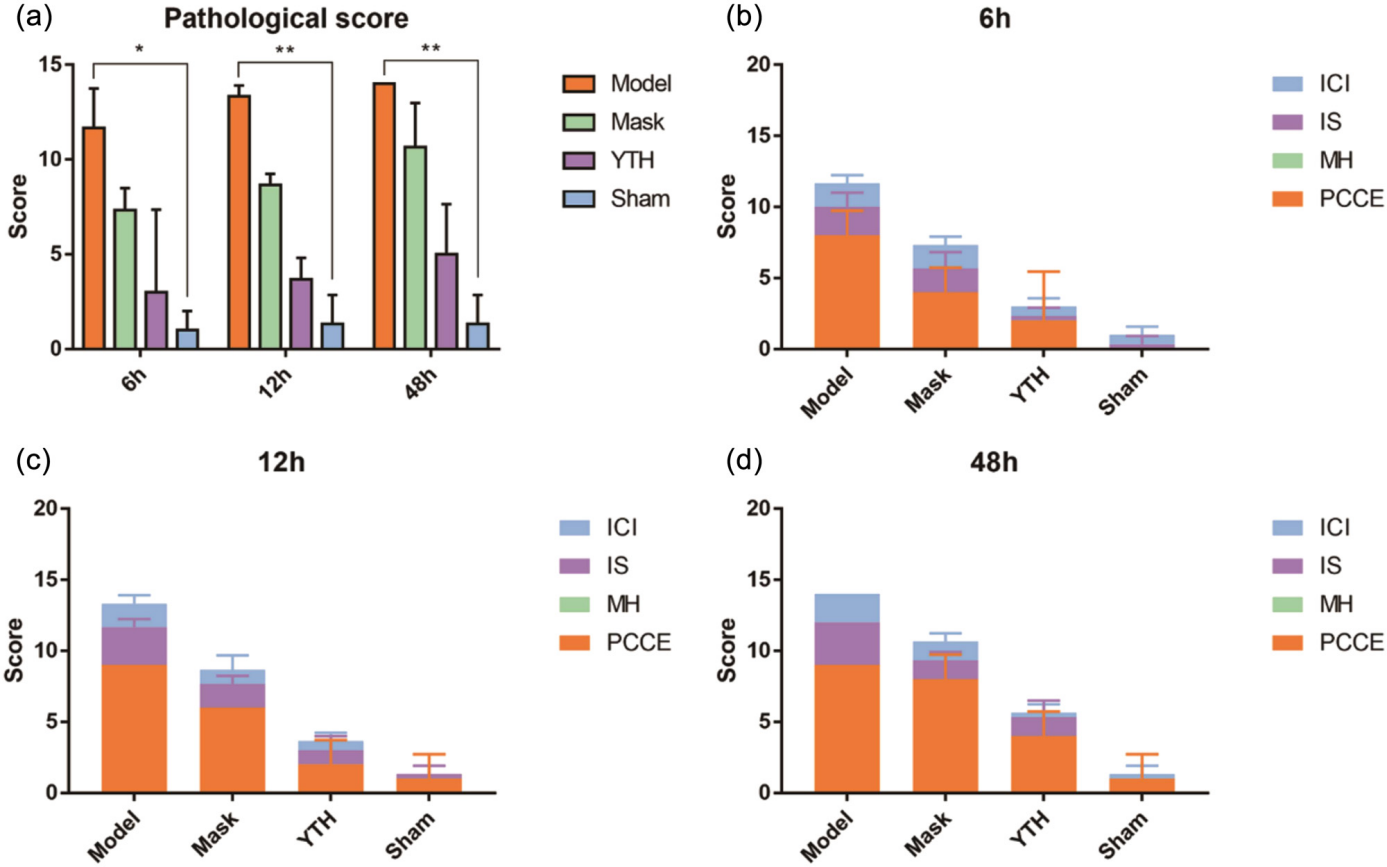
Tracheal pathology scores under different wetting methods. Changes in tracheal pathology scores in different groups at different time points. (a) The total pathology scores of each group at each time point. (b–d) The percentage of each part of the scores at each time point. Results are expressed as *x* ± *s* (*n* = 3). A rank sum test was used to compare the differences. *P < 0.05, **P < 0.01, ***P < 0.001.

We then collected tracheal tissues from each experimental group and performed HE staining to evaluate the effects of tracheal intubation and airway wetting on the trachea’s general morphology and changes in pathological scores. There were no significant pathological changes in the general trachea morphology in the Sham group. As seen in Figure 2, the Sham group had intact and clear pseudocomplex ciliated columnar epithelium without secretion in the tracheal lumen 6–48 h postoperatively; yet, a little infiltration of inflammatory cells was seen at 6 h, probably due to local irritation from the sham surgery. In contrast, the Model group had a disorganized tracheal tissue structure with exfoliated airway epithelial tissue, more secretions in the lumen, and a large infiltration of inflammatory cells in the subepithelial tissue (Figure 3), and had the highest pathological score among groups (Figure 4).

Mask and YTH device wetting reduced pathological scores, and the effect of YTH device wetting was more effective (Figure 4a). Mask and YTH wetting could protect the structural integrity of airway tissue and suppress the airway inflammatory response to varying degrees (Figure 3). For airway epithelium, the YTH device ensured the structural integrity of airway epithelial cells at 48 h. Mask wetting slowed airway epithelial cell exfoliation, although the airway epithelium in this group was damaged due to inadequate wetting at 48 h (Figures 3 and 4d). Also, the YTH device performed better at suppressing inflammatory cell aggregation and inflammatory response in the airway subepithelial tissue at 48 h and accounted for less weight in the pathologic score (Figure 4b–d). It was noteworthy that neither sham surgical manipulation nor two wetting methods resulted in glandular hyperplasia in the airway.

**Figure 5:**
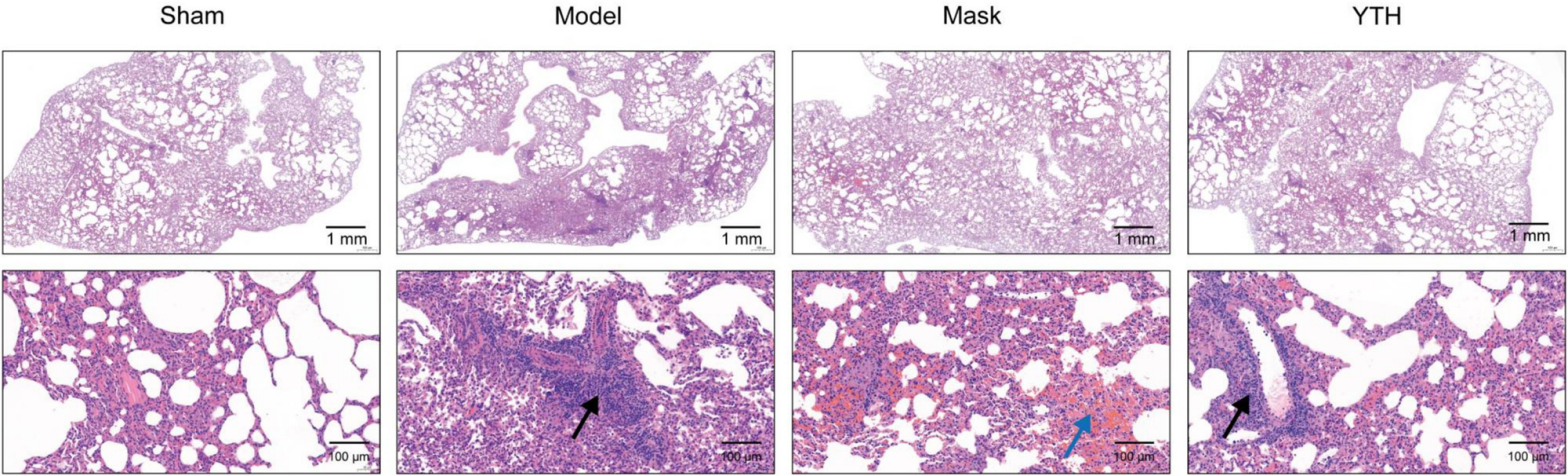
Representative HE staining images of lung tissue at 48 h. Representative image of lung tissue. Black arrows: infiltration of inflammatory cells; blue arrows: infiltration of erythrocytes.

Representative HE staining images of lung tissue at 48 h are shown in Figure 5. The lung tissue of rabbits in the Sham group showed normal morphology and structure. The lung tissue of rabbits in the Model group had blurred morphology and alveolar structure with inflammatory cell infiltration (shown by black arrows, Figure 5). The alveolar structure of rabbits in the Mask group was preserved after 48 h humidification; however, many erythrocytes were observed (shown by blue arrows, Figure 5). The lung tissue of rabbits in the YTH group maintained normal morphology with less infiltration of inflammatory cells.

## 4 Discussion

Long-term tracheotomy leads to loss of airway water due to the airway opening, resulting in patients’ dry airways, sticky sputum, and even sputum blockage, causing hypoxia and asphyxia, which poses a challenging issue in critical clinical care [21,22]. The airway mucosa exerts the protective function, requiring inhalation of moisture-saturated air. The degree of airway mucosal damage is directly proportional to the duration of ventilation without humidified gas [23]. The newly developed integrated device for continuous airway humidity and oxygen supply converts the wetting water in the collection cup into small atomized molecules through oxygen drive, reducing airway irritation. In this way, the device ensures oxygen supply and provides continuous airway humidity, avoiding respiratory water loss and airway blockade by sputum crust, conforming to the airway environment, increasing the utilization of the wetting solution, protecting tissue integrity, and inhibiting airway inflammation. The device is more stable, safe, and effective compared to other methods.

Foreign countries usually use active humidification equipment (MR850), HH, and artificial nose for humidification. However, the high cost of the MR850 humidifier and the airway-blocking effect of the artificial nose for patients with viscous sputum limit their clinical application. The common care method in China is the continuous airway drip method, which is more convenient and inexpensive. However, the wetting solution is easily deposited in the thicker trachea, leaving the bronchioles undetected. Besides, the connection of the wetting solution falls off easily. The external fixation tape, which is exposed to contamination and requires frequent replacement, increases the occurrence of cross-infection. In addition, the external mouth of the air vent may be partially closed by the tape, resulting in ventilation disorders. On the other hand, these procedures increase the workload of medical and nursing care [24]. To solve the practical clinical difficulties and reduce complications caused by inadequate and improper nursing operations, we developed an integrated airway wetting device as demonstrated in Figure S1 (Patent No. ZL201821869778.6). The device was more stable, calling for simple operations, eliminating the need for frequent care, and reducing staff workload. This study’s result suggests that the integrated device could provide better wetting efficiency under the same wetting conditions and provide stable wetting conditions in a shorter time. Compared to mask wetting, the integrated device could return to normal levels from pulmonary water loss within 12 h, preserving the integrity of airway epithelial tissue and reducing tissue damage in the early stage of tracheotomy. The underlying reason for the effect might be the higher utilization of the wetting solution of the integrated device at the same ventilation volume (4 L/min, SaO_2_ around 95% in each group). In this study, no mucus glands and cupped cell hyperplasia were observed in the trachea of the tracheotomy rabbit model within 48 h, which contradicted clinical observations [15]; this might be due to the relatively short observation time of 48 h compared to the clinical prolonged dry gas inhalation stimulation. We inferred that there could be more significant manifestations of glandular hyperplasia if the observation time was extended.

Inadequate humidification in tracheotomized patients results in poor sputum clearance and an increased possibility of infection. A humidifier, nebulizer, or passive humidifier can prevent sputum crust formation and reduce postoperative complications such as infection [25,26]. Respiratory cell injury due to dry gas triggers an inflammatory response, notably the release of inflammatory mediators, which is a systemic effect that can damage other tissues and organs [27]. As one of the inflammatory cytokines mainly produced by mononuclear macrophages, TNF-α is involved in various cellular immune responses, inducing elevated expression of pulmonary capillary adhesion molecules and the release of the inflammatory factor IL-8, which function as an important chemokine of neutrophils [28]. TNF-α also promotes mononuclear macrophages to produce IL-6 through Toll-like receptor-mediated signaling that activates the cis-regulatory element of IL-6 [29]. IL-8 and IL-6 promote inflammatory responses, and their elevated expression reflects the severity of inflammation [30]. In this study, water vapor loss due to an open airway caused desiccation damage to airway epithelial cells, resulting in local epithelial tissue necrosis, subcutaneous inflammatory cell infiltration, and elevated expression of circulating inflammatory factors. Rapid and adequate airway humidification suppressed the expression of pro-inflammatory factors in the serum after tracheotomy in the early stage, reducing the inflammatory response and local accumulation of inflammatory cells, thus protecting the normal function of the airway. Similar findings have been previously reported suggesting that adequate airway wetting inhibits TNF-α in the BALF [18] and serum TNF-α and IL-6 expression in the mechanically ventilated model [27]. IL-10 is an inflammatory suppressor produced by various immune cells, including macrophages, neutrophils, NK cells, and T cells. It inhibits the release of immune mediators, antigen expression, and phagocytosis [31,32]. IL-10 mainly activates the downstream JAK/STAT signaling pathway and regulates the expression of IL-10 effector genes [33]. In this study, we observed a partial increase in circulating IL-10 expression at 12 h of airway wetting, suggesting that airway wetting might participate in suppressing the inflammatory response by promoting IL-10 expression.

## 5 Conclusions

Our self-developed integrated wetting device can increase the utilization of wetting solutions, enhance the effect of wetting to protect tissue integrity, suppress airway inflammation, simplify nursing operation steps, and reduce labor waste and airway complications such as infection caused by human operation. It may be potentially used as a new method for clinical nursing practice.

Rapid and adequate humidification is important to ensure the structural integrity of the airway tissue and suppress the inflammatory response after tracheotomy. Recently, we have designed an anti-sputum spattering sputum collector (Patent No. 2018208423205). During the pandemic of COVID-19, the workload of nursing and the danger from aerosols were closely connected. In the future, we will extend the role of this airway humidity device function and further explore methods to avoid injury to healthcare workers by aerosol spraying during the airway care of tracheotomized non-mechanically ventilated patients, simplify nursing procedures, relieve clinical workload, and induce incidence of infection and hospitalization expense.

## Supplementary Material

Supplementary material
