# Supplementary material for: Application of the integrated airway humidification device enhances the humidification effect of the rabbit tracheotomy model
Source: Open Life Sci. 2024 Feb 8;19(1):20220825. doi: 10.1515/biol-2022-0825 (PMC10898622; doi:10.1515/biol-2022-0825)
Supplement: Supplementary material [file biol-2022-0825-sm.pdf]

Supplementary material

Supplementary Material 1. The mechanism of the integrated airway humidification device

An autogenous cutting oxygen humidification device, comprising: the tracheotomy tee joint is provided with a first pipe orifice serving as an inclined through pipe orifice, a second pipe orifice serving as a straight pipe orifice and a third pipe orifice; the medical atomizer is provided with a liquid inlet, a mist outlet and an air inlet; the two ends of the medical corrugated pipe are respectively connected with the first pipe orifice and the mist outlet; the infusion tube is connected with the liquid inlet; an oxygen pipe connected with the air inlet; a tracheotomy cannula or a tracheal cannula connected to the third tube orifice; the anti-splashing sputum collector is an open container, and a tracheotomy tee joint is arranged on the side wall of the open container. as shown in the Figure S1.

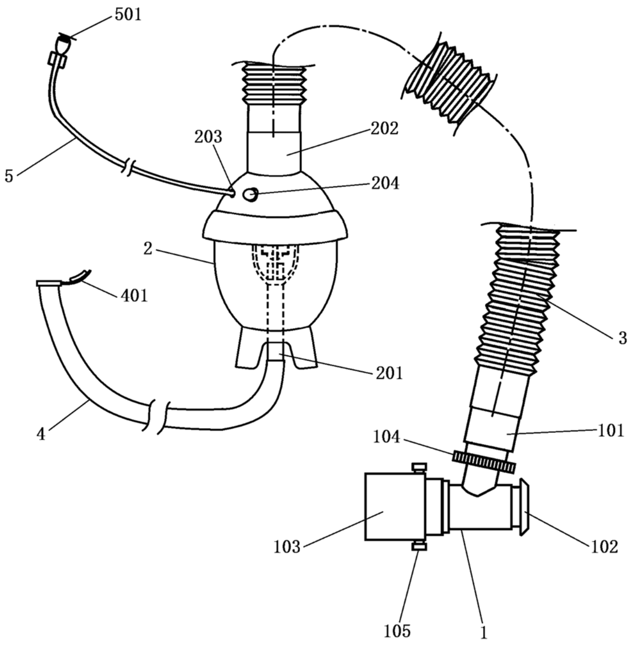

Figure S1: Integrated airway humidification device.
